# Finding an Effective Freezing Protocol for Turkey Semen: Benefits of Ficoll as Non-Permeant Cryoprotectant and 1:4 as Dilution Rate

**DOI:** 10.3390/ani10030421

**Published:** 2020-03-03

**Authors:** Michele Di Iorio, Giusy Rusco, Roberta Iampietro, Maria Antonietta Colonna, Luisa Zaniboni, Silvia Cerolini, Nicolaia Iaffaldano

**Affiliations:** 1 Department of Agricultural, Environmental and Food Sciences, University of Molise, 86100 Campobasso CB, Italy; michele.diiorio@unimol.it (M.D.I.); giusyrusco@gmail.com (G.R.); robertaiampietro@hotmail.it (R.I.); 2 Department of Agricultural and Environmental Science, University of Bari Aldo Moro, 70126 Bari BA, Italy; mariaantonietta.colonna@uniba.it; 3 Department of Veterinary Medicine, University of Milan, 20122 Milano MI, Italy; luisa.zaniboni@unimi.it

**Keywords:** *Meleagris gallopavo*, cryopreservation procedure, Ficoll, dilution rate, sperm cryobank

## Abstract

**Simple Summary:**

The most adopted biotechnology for the conservation of genetic resources in avian species is semen cryopreservation. Therefore, the identification of a reference cryopreservation procedure represents a key point for ensuring the long-term conservation of genetic diversity in birds, through the implementation of a semen cryobank. In this study, our goal was to discover an effective freezing protocol for *Meleagris gallopavo* in order to realize the first Italian semen cryobank of autochthonous chicken and turkey breeds within our project (TuBAvI). For this purpose, we investigated the effects of three non-permeant cryoprotectants (sucrose, trehalose, and Ficoll) and two dilution rates (1:2 and 1:4) on the in vitro cryosurvivability of turkey spermatozoa. After thawing, the best semen quality was found in semen frozen in the presence of Ficoll and diluted at a final rate of 1:4. This paper provides encouraging results, however further studies are programmed to standardize the semen cryopreservation protocol.

**Abstract:**

The present study aimed to find an effective cryopreservation protocol for turkey semen through the combined use of dimethylsulfoxide (DMSO) and three non-permeant cryoprotectants (NP-CPAs), sucrose, trehalose, and Ficoll 70. In addition, the action of two dilution rates (1:2 and 1:4) were also investigated. Semen was processed according to two final dilution rates and the following treatments: Tselutin extender (TE)/DMSO (control), TE/DMSO + sucrose or trehalose 50, 100, 200, or 400 mM, and TE/DMSO + Ficoll 0.5, 0.75, 1, or 1.5 mM. In total 26 different combinations treatments were achieved. The diluted semen was filled up into straws and frozen on liquid nitrogen vapor. The post-thawing sperm quality was assessed by analyzing motility, membrane integrity, osmotic resistance, and DNA integrity. The results obtained revealed a significant effect of NP-CPA concentration on total and progressive motility, on most of the kinetic parameters, on membrane integrity and DNA integrity, while the post-thaw quality was less affected by dilution rate. The highest post-thaw quality for all sperm quality parameters assessed except curvilinear velocity (VCL) and DNA integrity were found in semen frozen with 1 mM Ficoll/1:4 (*p* < 0.05). Our findings provide an important contribution for the identification of a reference procedure for turkey semen cryopreservation, in order to create the first national avian semen cryobank.

## 1. Introduction

In the last few decades in Italy, Farm Animal Genetic Resources (FAnGR) of avian species have rapidly declined, mainly due to the use of commercial hybrids in intensive breeding. The role and contribution of FAnGR has often been overlooked. Indigenous FAnGR carry genes that enable animals to tolerate harsh environments and repel the epidemic diseases from attacking [1,2,3]. Furthermore, preserving animal genetic resources will maintain our traditions and provide end users with multiple opportunities, including the enhancement of food quality and additional options to sustain economically on changing markets. The conservation of FAnGR, through the adoption of in situ and ex situ strategies, is an action undertaken to ensure the diversity of farm animal genetic material. In avian species, in addition to in vivo management, in vitro conservation is strategic in order to secure genetic diversity of a wide range of lines and breeds, and/or to contribute in creating new resources [4,5,6,7,8].

Semen cryopreservation is the most suitable technology for the ex situ in vitro conservation of avian genetic resources and preservation of rare breeds since it is the only non-invasive and the least expensive in vitro method available to date [3,9,10,11]. However, other methods within ex situ in vitro strategies including primordial germ cells and gonadic tissues technologies are still in progress but may be seen as methods that are complementary to semen cryopreservation [3,12,13,14]. Hence, the obtainment of an effective semen cryopreservation protocol in avian species represents a key point for ensuring the long-term conservation of genetic diversity through the creation of a semen cryobank. Different freezing procedures have been developed to cryopreserve avian semen [4,8,15,16,17,18,19]. Effective semen cryopreservation protocols have allowed the creation of semen cryobanks for various wild and some domestic chicken species [3,20,21,22,23,24]. However, up to day research efforts have not yet served in creating a turkey semen cryobank.

Recently, thanks to our financed project “TuBAvI” by the Ministry of Agricultural, Food and Forestry Policies (MiPAAF) the first Italian semen cryobank of autochthonous chicken and turkey breeds will be realized. The fundamental assumption for the creation of a semen cryobank is the development of a successful freezing protocol. Notice that the semen cryopreservation technique in avian species and particularly in *Meleagris gallopavo* is still not satisfactory because of the inability of turkey spermatozoa to successfully survive during the freezing/thawing process with obvious detrimental consequences on fertility [22,25,26,27,28]. This is due to the increased susceptibility of turkey spermatozoa to the damage that occurs during the freezing process compared to those of chicken [9,22,25,26,27,29]; for these reasons procedures developed to cryopreserve chicken semen are inefficient and thus not transferable to the turkey semen. Moreover, the obtainment of an effective semen cryopreservation protocol would have practical benefits for turkey production. Turkeys are the only commercial poultry species that depend entirely upon AI for fertile egg production.

Over the years the scientific community has been committed to discover an effective freezing protocol for turkey semen by studying the different variables that affect sperm cryosurvival: extender, dilution rate, cryoprotectant (CPA), freezing conditions, packaging system, and warming procedure [25,26,28,30,31,32,33]. The results deriving from these studies have given highly variable success rates; therefore, there is a clear need to standardize the whole freezing and thawing process to minimize variability in results. 

Among the studies undertaken, the choice of permeant (P) and non-permeant (NP) CPAs and in particular their combination gives interesting results but yet to be fully explored. It is known that the P- CPAs mainly involved in freezing protocols for turkey semen are glycerol, dimethylsulfoxide (DMSO), ethylene glycol, and dimethylacetamide (DMA) [9,25,26,27,28,32,33]. 

However, in order to reduce the cryo-damages caused by permeant CPAs, the addition of NP- CPAs in the freezing medium in combination with permeant ones have been considered in avian sperm [8,32,34,35]. The main NP-CPAs employed for semen cryopreservation in avian species have been PVP [3,4,36], sucrose, and trehalose [32,34], moreover, recently the Ficoll 70 has been used successfully in chicken [35] and dextran in turkey [37]. To the best of our knowledge there are only a few papers in the literature regarding the combination between P-CPAs and NP-CAPs in the freezing protocol for turkey semen and the use of Ficoll as a NP-CPA has not yet been tested in the turkey. In our previous paper [26] after the cooling phase, the semen was diluted with freezing extender containing as P-CPA the DMSO and to our knowledge there is scarce information about the effects of dilution rate. In this context, this study was designed to improve the freezing procedure for turkey semen by comparing Ficoll 70 with sucrose and trehalose, at different concentrations, on in vitro post-thaw semen quality. Moreover, two dilution rates were also evaluated. 

## 2. Materials and Methods

### 2.1. Experimental Design

The model used for the turkey semen cryopreservation was set up in a 3 × 4 × 2 design as follows: NP-CPA (sucrose, trehalose, or Ficoll), NP-CPA concentration (50, 100, 200, or 400 mM of sucrose or trehalose and 0.5, 0.75, 1, or 1.5 mM of Ficoll) dilution rate (1:2 or 1:4). Samples of pooled turkey semen were processed for freezing using the full combinations of these factors.

### 2.2. Chemicals

The LIVE/DEAD Sperm Viability Kit was purchased from Molecular Probes, Inc. (Eugene, OR, USA). All other reagents used in this study were obtained from Sigma, Chemical Co. (Milan, Italy).

### 2.3. Animals 

The animals used during this study were 150 turkey males of the Hybrid Large White line British United Turkeys (B.U.T.) supplied by Agricola Santo Stefano (Amadori Group, TE, Italy). Turkeys were reared in a poultry house in a controlled environment with artificial lighting (14 h light–10 h dark cycle) and given free access to a standard commercial feed and water. 

### 2.4. Extender Preparation

**Figure 1:**
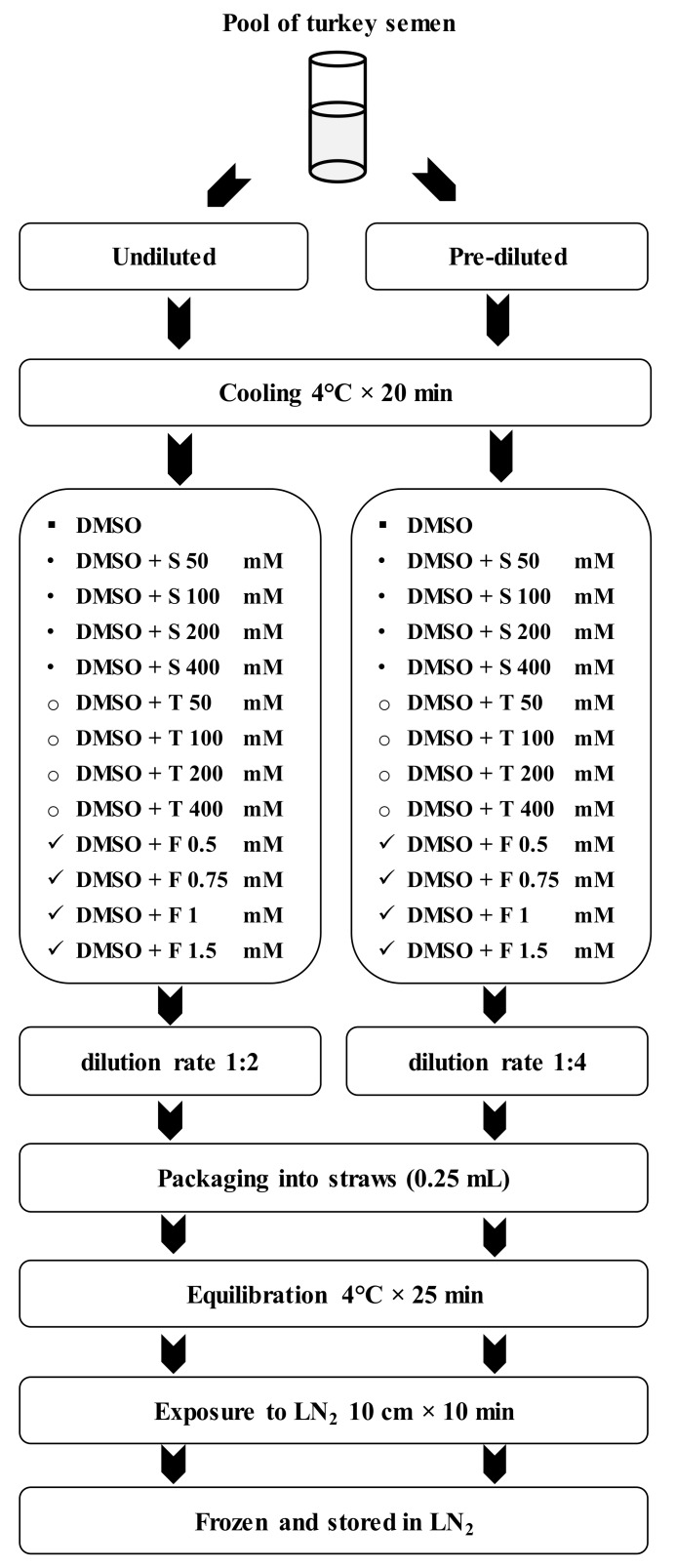
Flow diagram showing the different combinations of the steps of the freezing process rendering 26 different cryopreservation treatments. DMSO: dimethylsulfoxide; S: sucrose; T: trehalose; F: Ficoll; LN2: liquid nitrogen.

A base cryodiluent prepared at our laboratory was used. This extender is composed of Tselutin (Table 1) [38] containing 20% DMSO (v:v) as the P-CPA (control group). To this extender we added the NP- CPAs at four different concentrations: 50, 100, 200, or 400 mM for sucrose and trehalose and 0.5, 0.75, 1, or 1.5 mM for Ficoll to yield 13 different freezing extenders (Figure 1).

### 2.5. Semen Processing 

The experiments were carried out during the July–September 2018 period, corresponding to the best period of the reproductive cycle (32^nd^ − 44^th^ week) to produce semen with higher quality as show in our previous papers [39,40].

Semen was collected by abdominal massage and pooled (1 ejaculate/male; 16−20 ejaculates/pool) to avoid the effects of individual differences among males. A total of 8 pools were used, each pool contained at least 10 mL of semen. The quality of the fresh semen was assessed in an aliquot taken from each pool as described below. Each pool was split into two subsamples, one aliquot was pre- extended (1:1; v/v) with a Tselutin extender and the other one remained undiluted, each semen aliquot was subsequently cooled at 4 °C for 25 min.

After cooling, both semen subsamples (pre-diluted and undiluted) were divided into aliquots and diluted 1:1 (v:v) with the 13 freezing extenders respectively (Figure 1). Two final dilution rates were obtained: 1:2 (1 mL semen + 1 mL extender), and 1:4 (1 mL semen + 3 mL of extender) and in total 26 different combinations were achieved (Figure 1). 

Semen was aspirated into 0.25 mL plastic straws using a manual micro-aspirator (IMV- Technologies), the straws were marked with an alphanumeric code (to define the dilution rate and NP-CPA concentrations), and five different colors of straws were used (to mark the different CPAs). In total, 832 straws were used [4 straws × 26 treatments (13 freezing extender × 2 dilution rate) × 8 replicates]. Then, the straws were equilibrated at 4°C for 20 min. Semen was frozen by exposure to liquid nitrogen vapor 10 cm above the liquid nitrogen surface for 10 min [26], after this period the straws were plunged into liquid nitrogen (–196 °C) and were stored in a liquid nitrogen tank for at least three months before analysis. Sperm samples were thawed by immersing the straws in a water bath at 50°C for 10 seconds.

### 2.6. Assessment of Sperm Quality

In both the fresh and thawed semen samples, sperm motility, membrane integrity, functional integrity of the sperm membrane, and DNA integrity were determined in duplicate. Sperm motility was evaluated by means of a computer-aided sperm analysis (CASA) system connected to a phase contrast microscope (Nikon Eclipse model 50i; negative contrast) using the Sperm Class Analyzer (SCA) software (version 4.0, Microptic S.L., Barcelona, Spain). Each semen sample of fresh or frozen semen was extended in 0.9% NaCl to reach a sperm concentration of 100 × 10^6^/mL and incubated for 5 min at 38 °C. Then, 5 µL of semen was placed on a microscope slide and observed under the microscope. The sperm motion parameters recorded were: motile spermatozoa (%), progressive motile spermatozoa (%), curvilinear velocity (VCL, (µm/s)), straight-line velocity (VSL, (µm/s)), average path velocity (VAP, (µm/s)), linearity (LIN, (%)) and straightness (STR, (%)). At least 1000 sperm tracks in five microscopic fields for each sample were assessed at 100× total magnification.

Membrane integrity of spermatozoa was determined using fluorescent stains SYBR-14 and propidium iodide (PI) as previously described [26]. This procedure was performed by mixing 5 μL of semen with 80 μL of Tselutin extender and 2 μL SYBR-14 (diluted 1:100 in DMSO). The extended semen was then incubated at 38 °C for 10 min, and 5 μL propidium iodide (diluted 1:100 in PBS) were added followed by incubation at 38 °C for a further 5 min. Next, 5 µL of this sample was deposited onto microscope slides and viable/non-viable spermatozoa were detected with a fluorescence microscopy (Leica Aristoplan; Leitz Wetzlar, Heidelberg, Germany; blue excitation filter λ = 488 nm; × 100 oil immersion objective; total magnification × 1000). SYBR-14 is a membrane-permeable DNA stain for live spermatozoa producing bright green fluorescence of nuclei. Propidium iodide stains the nuclei of membrane-damaged cells red, so that spermatozoa showing green fluorescence are recorded as live and those fluorescing red as dead. After counting minimum 200 spermatozoa, percentages of viable spermatozoa were calculated at the ratio: green cells/(green cells + red cells) × 100.

To determine the functional integrity of the sperm membrane, a hypo-osmotic swelling test (HOST) was used [25,26]. Aliquots of 5 µL of diluted semen were added to 80 µL of distilled H_2_O and then stained with SYBR and PI and counted as described above for sperm membrane integrity. This test is effective for assessing the percentage of viable spermatozoa that are capable of withstanding hypo-osmotic stress in vitro. Only SYBR-14 can penetrate osmotic-resistant sperm cells and these appear green on fluorescence microscopy. Conversely, damaged membranes permit the passage of PI, staining spermatozoa that have lost their functional integrity red.

Sperm DNA integrity was evaluated using an acridine orange (AO) test as described by Gandini et al. [41]. We adapted this test following the procedure used for rabbit semen [42,43] with some minor adaptation. Specifically, 5 µL of fresh or thawed semen was extended with 100 µL of Tselutin extender. Then, 10 µL of this solution was smeared onto a microscope slide and fixed for at least 10 h in a 3:1 methanol: glacial acetic acid solution. Smears were then stained with an AO solution (0.2 mg/mL in water) in the dark at room temperature for 5 min; subsequently, the mounted slide was examined using a fluorescence microscope with a 490 nm excitation light and 530 nm barrier filter, minimum 200 spermatozoa per slide were counted and scored as possessing green or yellow- orange-red fluorescence (intact DNA or damaged DNA, respectively), and the percentage of DNA integrity was calculated.

### 2.7. Statistical Analysis

To compare the different treatments, we used a randomized block design in a two by four factorial arrangement (two dilution rate × four NP-CPA concentration), for each NP-CPA tested (sucrose, trehalose, and Ficoll) with eight replicates per treatment.

Sperm variables (CASA motility parameters, membrane integrity, osmotic resistance and DNA integrity) were compared among the treatments by ANOVA followed by Duncan’s comparison test. A generalized linear model procedure was then used to determine the fixed effects of dilution rate and NP-CPA concentration and their interactions on the sperm quality variables. Significance was set at *p* < 0.05. All statistical tests were performed using SPSS software (SPSS 15.0 for Windows, 2006; SPSS, Chicago, IL, USA).

## 3. Results 

The semen parameters recorded in fresh semen are shown in Table 2, these data indicated its good initial quality. The post-thaw semen quality variables (motility parameters, membrane integrity, osmotic resistance, and DNA integrity) recorded in the DMSO, sucrose, trehalose, and Ficoll groups are provided in Table 3, Table 4, Table 5 and Table 6 respectively. No significant differences were observed for all sperm variables assessed in semen frozen in the presence of only DMSO considering the two dilution rates (Table 3). 

The sucrose concentration significantly affected all sperm parameters except LIN and STR whilst the effect of the dilution rate was significant only for STR and osmotic resistance. No significant interaction effect of concentration × dilution rate was observed (Table 4). Higher total motility, progressive sperm motility, VCL, VSL, and VAP were recorded in semen frozen using the combination of 50 mM of sucrose/dilution rate of 1:2, this resulted as significant in respect to 400 mM for both the dilution rate and the treatment 100 mM/1:2 for the total motility (Table 4). 

A significant effect of trehalose concentration was detected in all sperm variables except for LIN, STR, and osmotic resistance. While the dilution rate was significant along the following parameters: total motility, VSL, VAP, LIN, STR, sperm membrane integrity, and DNA integrity. Better post-thaw semen quality was recorded using a dilution rate of 1:4 and 100 mM of trehalose, registering a significant difference in respect to 200 and 400 mM in both dilution rates for total motility, progressive motility, and DNA integrity whilst for sperm membrane integrity a significant effect was found compared to the 400 mM/1:2 or 1:4 (Table 5).

The fixed effects of NP-CPA concentration, dilution rate, and their interactions on sperm quality variables for the Ficoll freezing protocol are shown in Table 6. Significant effects of NP-CPA concentration were detected on total and progressive motility, VSL, membrane integrity, and DNA integrity, the effect of dilution rate resulted significant only for LIN, STR, and membrane integrity. A significant effect of the interaction NP-CPA concentration × dilution rate was produced for the total and progressive motility.

The higher total motility values (*p* < 0.05) were recorded for the combination treatment 1 mM of Ficoll and dilution rate 1:4, the differences were not significant with the treatments 0.75 mM/1:2 or 1:4. Higher progressive motility percentages (*p* < 0.05) were observed also for the treatment 1 mM Ficoll/1:4 with no significant differences with respect to 0.5, 0.75, and 1 mM/1:2. Moreover, membrane integrity, VSL, and LIN parameters were significantly higher in the treatment 1 mM Ficoll/1:4 compared to all other treatments, except for membrane integrity with treatment 0.75 mM/1:4.

**Figure 2:**
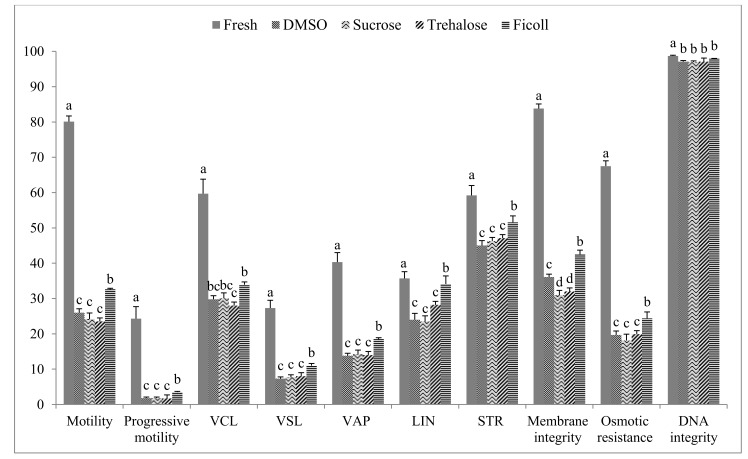
Comparison of semen quality measured in fresh and frozen/thawed semen considering the best freezing protocol identified for each CPA tested (N=8).^a–d^ Different superscripts letters within each semen parameter indicate a significant difference (*p* < 0.05). Motility (%); Progressive motility (%); VCL: curvilinear velocity (µm/s); VSL: straight-line velocity (µm/s); VAP: average path velocity (µm/s); LIN: linearity (%); STR: straightness (%); Membrane integrity (%); Osmotic resistance (%); DNA integrity (%).

However, in Figure 2, we compared the semen quality parameters of the fresh semen and those from the best freezing protocol identified for each CPA tested. All the sperm quality parameters significantly decreased after the freezing-thawing process. Moreover, a significant effect of the CPA type was reported. The highest values of total and progressive motility, VSL, VAP, LIN, STR, membrane integrity, and osmotic resistance (*p* < 0.05) were recorded using the cryodiluent containing 1 mM of Ficoll.

## 4. Discussion

Obtaining an effective turkey semen cryopreservation protocol is an important goal, because sperm cryopreservation is the most adapt technology to develop ex situ in vitro conservation programs of avian genetic resources [10,11,44]. So far, the semen cryopreservation in avian species is the only technology available to set up ex situ conservation programs because of the characteristics of the megalecithal egg [9,10,11,22]. 

Here the identification of a reference turkey semen cryopreservation protocol represents a milestone of our financed project and provides an important contribution for the creation of the first semen cryobank of autochthonous turkey breeds. From the results obtained, it emerged that among the 26 treatments tested the combination of Ficoll 1 mM and the dilution rate 1:4 resulted as the best freezing protocol for in vitro survivability of turkey spermatozoa. This freezing protocol returned as recovery rates (value in frozen semen/value in fresh semen × 100) of 40.7% for total sperm motility, 14.4% for progressive motility, 50.7% for membrane integrity, 36.1% for osmotic resistance, and 99.2% for DNA integrity.

Our in vitro results clearly revealed that the dilution rate significantly affected the post-thaw quality of turkey semen in a different way for each NP-CPA tested. The dilution rate of 1:4 was found to be more effective than the 1:2 ratio independently by the type and concentration of NP-CPA used. However, a foremost effect of 1:4 dilution rate was found in the treatment with trehalose for almost all of the parameters assessed. Concordantly with Iaffaldano et al. [25] the positive effect of the higher dilution rate (1:4) in respect to that of 1:2 is probably due to the different number of spermatozoa per mL of seminal plasma and/or freezing extender. This could be attributed to a different amount of DMSO per sperm cell or to the lower content of seminal plasma in the total volume of sperm sample. In this regard, previous researches showed that the seminal plasma seems to negatively affect turkey spermatozoa during both liquid storage and cryopreservation [25,45,46]. In effect, improved fertility rates have been obtained by removing the seminal plasma from turkey spermatozoa before freezing, as reviewed by Blesbois [22]. This finding suggests that in order to further improve the cryopreservation protocol in turkey semen, it would be necessary to standardize the optimal sperm concentration before the addition of CPAs. This will be our next goal.

Another point that emerged in this research was that the concentration of sucrose, trehalose, and Ficoll used here significantly influenced the most post thawing parameters; 100 mM of trehalose and 50 mM of sucrose worked better than the others concentration tested (200 and 400 mM) and finally 1 mM of Ficoll cryoprotected better in respect to the other concentrations studied. This is consistent with that reported in the literature; in fact, the beneficial effect of sugar as NP-CPAs depends on their concentration [47,48].

Unexpectedly, the addition of trehalose and sucrose, at all concentration used, did not improve the sperm cryosurvival compared to DMSO alone. In fact, combined treatments such as sucrose 50 mM/1:2 and trehalose 100 mM/1:4 showed similar post-thaw quality with semen frozen in the presence of only DMSO, instead the concentrations of 200 mM and 400 mM of both sucrose and trehalose caused an even more drastic reduction of post-thawing sperm quality. 

The harmful effect of higher concentrations (200 and 400 mM) of sucrose and trehalose could be due to the increased osmolarity of the extender that generates aggressive dehydration. Hence, this is caused by great and unneeded osmotic effect due to high external cryoprotectant concentration, which results in being deleterious to the sperm membranes as Thananurak et al. [8] also reported. Avian sperm contains a relatively low amount of intracellular water compared to mammalian spermatozoa, therefore, it is not necessary to induce a high level of dehydration during the freezing process, and thus high concentrations of NP-CAPs are not needed [8].

Ours results on the use of sucrose or trehalose are in discordance with those reported in the literature [34]. This could be related to the fact that different avian species and freezing protocols (concentrations of NP-CPA and P-CPA dissimilar) were used. Here, the rationale to test different concentrations of NP-CPAs was driven only by scarce information about the combination between DMSO and trehalose or sucrose and also by the absence of knowledge on the use of Ficoll in addition to DMSO.

The NP-CAPs used at similar concentration to the P-CPAs are less toxic and they explicate various protective actions, such as lowering the freezing temperature of the medium, reducing ice crystal growth and helping the sperm to stabilize the internal solute concentrations under osmotic stress [26,32,34,42,49]. In this regard, different external CPAs have already been tested in combination with P-CPAs to improve sperm cryosurvival in various mammalian [42,50,51,52,53] and avian species [32,34,35]. Specifically, in avian species, different sugars, mainly disaccharides [32,34,35,54] and also trisaccharides [3] have been used as NP-CPAs in combination with the P-CPA. Besides sugars, other substances have also been used as NP-CPAs, such as betaine hydrochloride in turkey [32]; PVP [3,4,36], glycine, and Ficoll in chicken [35]. 

In this study, among the NP-CPAs tested only the addition of Ficoll 1 mM significantly improved the post-thaw semen quality compared to DMSO alone. Moreover, the Ficoll 1 mM also returned significantly higher post-thaw semen quality than all treatments with trehalose, sucrose and DMSO alone. This finding is consistent with previous ones carried out in rabbit [55] and in chicken [35], that reported beneficial effects of Ficoll compared to other NP-CPAs. Ficoll 70 is a macromolecule; it shows up as a highly compact spherical polysucrose [56], which acts as an NP-CPA thanks to its ability to affect the viscosity of the cryopreservation solution and to preserve the cells during freezing/thawing [57,58,59]. Ficoll 70 has been previously used successfully for cryopreservation of different cells type such as rabbit morula and blastocyst stage embryos [60,61,62], human and mouse zonae pellucidae and embryos [57], equine embryo [63], and recently also for sperm cells [35,55].

The effectiveness of Ficoll as external CPA could be attributed mainly to its ability to affect the solution viscosity, guaranteeing thus a greater stability of the sperm plasma membrane, reducing mechanical deformations. In accordance with other authors, Ficoll leads to an adequate cellular dehydration, which is reflected in a reduction of ice crystal growth therefore resulting in a better ability of sperm cells to survive during the cryopreservation process [35,55]. Moreover, recently it has been shown that Ficoll 70 is efficient in minimizing the recrystallization process during the storage and warming of cells at non-cryogenic temperatures (−10 °C to −80 °C range) by preventing the water molecules from approaching the ice nuclei, and simultaneously lowering the activities of the water molecules [56]. Therefore, this biophysical mechanism could even occur at cryogenic temperatures of −196 °C.

In addition, in accordance with Gloria et al. [37], we assume that the smaller molecules such as sucrose and trehalose could enter the intra-cytoplasmic compartment of spermatozoa, interfering with sperm metabolism or structural integrity, while larger molecules such as Ficoll (70,000 MW) would not be transported into this compartment.

## 5. Conclusions

In conclusion our findings indicated that the turkey semen cryosurvival depends on the kind of NP-CPA used and its concentration.

The procurement of an effective semen cryopreservation protocol provides an important base for the implementation of the first national avian genetic resources semen cryobank in the TuBAvI project. Further studies are planned in order to find the optimal sperm concentration/straw so the cryopreservation protocol can be standardized better and also an evaluation of reproductive performances is needed to corroborate the efficacy of the frozen semen protocol.

These next challenges aiming to find an efficient, standardized freezing protocol for turkey semen could also improve the current prospects for the commercial use of frozen semen for the turkey breeding industry based entirely upon AI for fertile egg production.

## Figures and Tables

**Table 1:** Chemical composition of Tselutin extender.

Components	mM
Glucose	44.4
Sodium glutamate	128.0
Di-Potassium hydrogen phosphate	20.0
Magnesium acetate	7.0
Glycine	13.3
Glutamic acid	7.68
Inositol	11.1

**Table 2:** Sperm quality parameters (mean ± S.E.M.) measured in freshly collected turkey semen (N = 8).

MOT.%	MOT. PROG. %	VCL (µm/s)	VSL (µm/s)	VAP (µm/s)	LIN (%)	STR (%)	Membrane Integrity (%)	Osmotic Resistance (%)	DNA Integrity (%)	Sperm Concentration × 10^9^
80.1 ± 1.6	24.3 ± 3.4	59.7 ± 4.1	27.3 ± 2.2	40.3 ± 2.7	35.7 ± 1.9	59.2 ± 2.8	83.8 ± 1.3	67.5 ± 1.5	98.7 ± 0.2	8.9 ± 0.4

MOT: total motility; MOT PROG: progressive motility; VCL: curvilinear velocity; VSL: straight-line velocity; VAP: average path velocity; LIN: linearity; STR: straightness.

**Table 3:** Sperm quality variables (mean ± S.E.M.) recorded in turkey semen frozen in the presence of DMSO according to two dilution rates (N = 8).

Semen Treatment		Sperm Parameters									
	Dilution rate	MOT. %	MOT. PROG. %	VCL (µm/s)	VSL (µm/s)	VAP (µm/s)	LIN (%)	STR (%)	Membrane integrity (%)	Osmotic resistance (%)	DNA integrity (%)
DMSO	1:2	25.4 ± 1.0	1.8 ± 0.2	31.4 ± 0.8	7.1 ± 0.5	14.1 ± 0.5	21.1 ± 1.1	41.8 ± 0.8	34.8 ± 0.8	19.1 ± 1.4	96.8 ± 0.3
DMSO	1:4	26.0 ± 1.1	1.9 ± 0.3	29.8 ± 1.0	7.3 ± 0.5	13.8 ± 0.7	24.0 ± 1.8	45.0 ± 1.4	36.1 ± 0.8	19.7 ± 1.1	97.1 ± 0.3

MOT: total motility; MOT PROG: progressive motility; VCL: curvilinear velocity; VSL: straight-line velocity; VAP: average path velocity; LIN: linearity; STR: straightness.

**Table 4:** Sperm quality parameters (mean ± S.E.M.) measured in turkey semen frozen in the presence of sucrose according to the different concentrations and two dilution rates (N = 8).

Semen Treatment		Sperm Parameters									
NP-CPA Concentration	Dilution rate	MOT. %	MOT. PROG. %	VCL (µm/s)	VSL (µm/s)	VAP (µm/s)	LIN (%)	STR (%)	Membrane Integrity (%)	Osmotic Resistance (%)	DNA Integrity (%)
50 mM	1:2	24.2 ± 1.7^a^	1.8 ± 0.3^a^	30.1 ± 1.5^a^	7.7 ± 0.7^a^	14.3 ± 1.1^a^	23.5 ± 1.6^abc^	46.3 ± 1.0^b-e^	31.0 ± 1.3^ab^	18.1 ± 1.8^abc^	97.0 ± 0.3^ab^
100 mM	1:2	19.6 ± 1.9^b^	1.3 ± 0.4^ab^	28.3 ± 2.8^a^	5.8 ± 0.7^ab^	12.0 ± 1.0^ab^	18.6 ± 1.5^c^	41.9 ± 1.5^e^	31.2 ± 1.4^ab^	17.5 ± 1.7^abc^	96.5 ± 0.2^bc^
200 mM	1:2	22.1 ± 1.7^ab^	1.2 ± 0.3^ab^	28.3 ± 2.1^a^	6.8 ± 0.9^a^	12.4 ± 1.1^ab^	24.0 ± 2.1^abc^	46.1± 1.3^c-e^	29.2 ± 2.0^b^	17.0 ± 1.3^abc^	96.0 ± 0.3^cd^
400 mM	1:2	13.0 ± 0.4^c^	0.7 ± 0.2^c^	21.2 ± 1.3^b^	4.5 ± 0.4^b^	8.2 ± 0.4^c^	20.6 ± 2.0^bc^	47.4 ± 1.4^a^	22.3 ± 1.4^d^	14.7 ± 0.9^c^	95.5 ± 0.2^d^
50 mM	1:4	20.8 ± 1.0^ab^	1.3 ± 0.2^ab^	27.6 ± 1.3^a^	6.6 ± 0.4^ab^	13.2 ± 0.8^a^	24.7 ± 2.0^abc^	44.2 ± 1.0^c-e^	28.5 ± 2.1^bc^	19.2 ± 1.3^ab^	96.9 ± 0.3^ab^
100 mM	1:4	22.9 ± 1.1^ab^	1.6 ± 0.3^a^	28.5 ± 1.6^a^	7.3 ± 0.8^a^	13.6 ± 1.1^a^	27.0 ± 2.9^ab^	46.7 ± 2.2^ab^	34.7 ± 1.2^a^	20.7 ± 1.8^a^	97.3 ± 0.2^a^
200 mM	1:4	20.5 ± 1.2^ab^	1.4 ± 0.1^ab^	27.4 ± 1.5^a^	7.6 ± 0.5^a^	13.3 ± 0.8^a^	29.9 ± 1.5^a^	48.5 ± 1.1^a^	31.3 ± 0.8^ab^	19.8 ± 0.8^ab^	96.5 ± 0.2^bc^
400 mM	1:4	15.8 ± 1.0^c^	0.6 ± 0.2^c^	21.5 ± 1.0^b^	6.2 ± 1.0^ab^	10.2 ± 1.1^bc^	24.3 ± 4.2^abc^	47.6 ± 2.5^a^	24.8 ± 1.3^cd^	16.2 ± 0.5^bc^	96.5 ± 0.1^bc^
Concentration effect		*p* = 0.000	*p* = 0.002	*p* = 0.000	*p* = 0.035	*p* = 0.000	*p* = 0.258	*p* = 0.146	*p* = 0.000	*p* = 0.034	*p* = 0.000
Dilution rate effect		*p* = 0.755	*p* = 0.941	*p* = 0.498	*p* = 0.215	*p* = 0.156	*p* = 0.258	*p* = 0.009	*p* = 0.187	*p* = 0.027	*p* = 0.080
Concentration × dilution		*p* = 0.129	*p* = 0.463	*p* = 0.787	*p* = 0.363	*p* = 0.212	*p* = 0.182	*p* = 0.524	*p* = 0.201	*p* = 0.848	*p* = 0.053

^a–e^ Values within a column reporting different superscript letter differ significantly at *p* < 0.05. MOT: total motility; MOT PROG: progressive motility; VCL: curvilinear velocity; VSL: straight-line velocity; VAP: average path velocity; LIN: linearity; STR: straightness.

**Table 5:** Sperm quality parameters (mean ± S.E.M.) outcomes in turkey semen frozen with trehalose considering different concentrations and two dilution rates (N = 8).

Semen Treatment		Sperm Parameters									
NP-CPA Concentration	Dilution rate	MOT. %	MOT. PROG. %	VCL (µm/s)	VSL (µm/s)	VAP (µm/s)	LIN (%)	STR (%)	Membrane Integrity (%)	Osmotic Resistance (%)	DNA Integrity (%)
50 mM	1:2	21.1 ± 2.2^ab^	1.6 ± 0.5^ab^	29.4 ± 2.5^a^	6.8 ± 0.9^abc^	13.2 ± 1.6^ab^	20.9 ± 2.0^ab^	44.3 ± 1.1^ab^	29.2 ± 1.7^ab^	16.8 ± 1.4^a^	96.8 ± 0.2^ab^
100 mM	1:2	18.7 ± 1.7^bc^	1.5 ± 0.3^ab^	27.9 ± 2.4^a^	5.4 ± 0.9^ab^	10.9 ± 1.5^abc^	18.1 ± 1.6^b^	42.2 ± 0.9^ab^	30.6 ± 1.9^a^	18.5 ± 1.4^a^	96.9 ± 0.1^ab^
200 mM	1:2	16.7 ± 1.6^cd^	0.9 ± 0.1^bc^	23.7 ± 1.2^a^	4.9 ± 0.6^a^	9.8 ± 0.9^bc^	17.7 ± 2.0^b^	40.9 ± 1.7^b^	27.5 ± 1.8^ab^	16.9 ± 1.9^a^	95.9 ± 0.3^c^
400 mM	1:2	13.3 ± 0.9^d^	0.5 ± 0.1^c^	20.0 ± 1.5^b^	4.3 ± 0.7^b^	8.1 ± 1.1^c^	18.1 ± 2.4^b^	45.6 ± 1.7^ab^	21.9 ± 1.8^c^	17.1 ± 1.3^a^	95.1 ± 0.5^d^
50 mM	1:4	21.6 ± 1.2^ab^	1.5 ± 0.2^ab^	29.2 ± 1.8^a^	7.6 ± 0.7^ab^	13.7 ± 1.1^a^	26.0 ± 2.9^a^	46.3 ± 2.1^ab^	31.9 ± 1.1^a^	19.1 ± 1.3^a^	96.9 ± 0.2^ab^
100 mM	1:4	23.5 ± 1.1^a^	1.7 ± 0.3^a^	28.0 ± 1.9^a^	8.0 ± 1.0^a^	14.0 ± 1.4^a^	28.2 ± 1.5^a^	47.1 ± 0.8^a^	32.0 ± 1.4^a^	19.9 ± 0.6^a^	97.1 ± 0.2^a^
200 mM	1:4	18.6 ± 0.9^bc^	1.0 ± 0.2^bc^	25.4 ± 1.6^a^	6.7 ± 0.7^abc^	11.8 ± 0.8^abc^	27.0 ± 3.0^a^	47.1 ± 2.4^a^	30.9 ± 1.5^a^	19.1 ± 0.9^a^	96.6 ± 0.2^abc^
400 mM	1:4	14.2 ± 0.9^d^	0.5 ± 0.1^c^	24.9 ± 1.2^b^	5.6 ± 0.6^abc^	11.0 ± 1.1^abc^	22.9 ± 2.7^ab^	45.9 ± 1.9^ab^	25.2 ± 1.3^bc^	17.6 ± 1.4^a^	96.1 ± 0.2^bc^
Concentration effect		*p* = 0.000	*p* = 0.000	*p* = 0.002	*p* = 0.039	*p* = 0.013	*p* = 0.417	*p* = 0.613	*p* = 0.000	*p* = 0.573	*p* = 0.000
Dilution rate effect		*p* = 0.037	*p* = 0.999	*p* = 0.212	*p* = 0.006	*p* = 0.017	*p* = 0.000	*p* = 0.008	*p* = 0.017	*p* = 0.090	*p* = 0.009
Concentration × dilution		*p* = 0.366	*p* = 0.796	*p* = 0.473	*p* = 0.720	*p* = 0.711	*p* = 0.824	*p* = 0.375	*p* = 0.899	*p* = 0.894	*p* = 0.336

^a–d^ Values within a column reporting different superscript letter differ significantly at *p* < 0.05. MOT: total motility; MOT PROG: progressive motility; VCL: curvilinear velocity; VSL: straight-line velocity; VAP: average path velocity; LIN: linearity; STR: straightness.

**Table 6:** Sperm quality variables (mean ± S.E.M.) obtained in turkey semen frozen in the presence of Ficoll considering different concentrations and two dilution rates (N = 8).

Semen Treatment		Sperm Parameters									
NP-CPA Concentration	Dilution rate	MOT. %	MOT. PROG. %	VCL (µm/s)	VSL (µm/s)	VAP (µm/s)	LIN (%)	STR (%)	Membrane Integrity (%)	Osmotic Resistance (%)	DNA Integrity (%)
0.5 mM	1:2	26.9 ± 1.1^bcd^	2.8 ± 0.3^abc^	33.3 ± 1.3^ab^	8.5 ± 0.5^b^	15.8 ± 0.7^abc^	24.0 ± 1.1^b^	45.3 ± 0.9^c^	34.1 ± 1.5^c^	19.1 ± 2.0^a^	97.4 ± 0.3^bc^
0.75 mM	1:2	30.7 ± 2.2^ab^	3.0 ± 0.4^ab^	34.0 ± 1.3^a^	8.5 ± 0.4^b^	16.3 ± 0.8^abc^	24.2 ± 0.9^b^	44.7 ± 0.7^c^	37.1 ± 1.8^bc^	22.4 ± 2.4^a^	97.8 ± 0.2^ab^
1 mM	1:2	29.0 ± 1.6^bc^	2.7 ± 0.3^abc^	33.0 ± 1.3^ab^	8.9 ± 0.6^b^	16.2 ± 0.9^abc^	24.3 ± 1.6^b^	45.4 ± 0.9^c^	36.0 ± 1.7^bc^	21.4 ± 1.9^a^	97.5 ± 0.2^abc^
1.5 mM	1:2	23.5 ± 1.7^de^	1.9 ± 0.3^cd^	30.0 ± 1.9^ab^	8.0 ± 0.9^b^	14.4 ± 1.3^bc^	24.2 ± 1.9^b^	46.5 ± 1.3^bc^	36.0 ± 1.0^bc^	19.8 ± 1.5^a^	96.7 ± 0.1^d^
0.5 mM	1:4	25.1 ± 0.8^cde^	1.8 ± 0.3^d^	29.3 ± 1.4^b^	7.7 ± 0.2^b^	13.7 ± 0.4^c^	28.5 ± 1.3^b^	49.5 ± 0.9^ab^	37.1 ± 1.2^bc^	23.1 ± 1.7^a^	97.1 ± 0.1^cd^
0.75 mM	1:4	30.8 ± 0.6^ab^	2.4 ± 0.2^bcd^	32.8 ± 0.6^ab^	8.8 ± 0.1^b^	17.5 ± 1.6^ab^	26.9 ± 0.8^b^	48.0 ± 0.9^abc^	40.5 ± 0.9^ab^	23.9 ± 1.9^a^	97.8 ± 0.2^ab^
1 mM	1:4	32.6 ± 0.3^a^	3.5 ± 0.2^a^	33.9 ± 0.8^a^	11.0 ± 0.6^a^	18.5 ± 0.4^a^	34.1 ± 2.3^a^	51.6 ± 1.8^a^	42.5 ± 1.2^a^	24.4 ± 1.8^a^	97.9 ± 0.1^a^
1.5 mM	1:4	22.1 ± 1.4^e^	1.6 ± 0.1^d^	31.2 ± 1.2^ab^	7.8 ± 0.9^b^	15.5 ± 1.8^abc^	27.0 ± 2.6^b^	48.3 ± 1.8^abc^	35.7 ± 1.9^c^	22.3 ± 1.5^a^	96.6 ± 0.1^d^
Concentration effect		*p* = 0.000	*p* = 0.000	*p* = 0.059	*p* = 0.005	*p* = 0.052	*p* = 0.123	*p* = 0.431	*p* = 0.024	*p* = 0.534	*p* = 0.000
Dilution rate effect		*p* = 0.510	*p* = 0.198	*p* = 0.378	*p* = 0.435	*p* = 0.453	*p* = 0.000	*p* = 0.000	*p* = 0.004	*p* = 0.073	*p* = 0.735
Concentration × dilution		*p* = 0.034	*p* = 0.013	*p* = 0.162	*p* = 0.276	*p* = 0.099	*p* = 0.152	*p* = 0.391	*p* = 0.158	*p* = 0.931	*p* = 0.112

^a–e^ Values within a column reporting different superscript letter differ significantly at *p* < 0.05. MOT: total motility; MOT PROG: progressive motility; VCL: curvilinear velocity; VSL: straight-line velocity; VAP: average path velocity; LIN: linearity; STR: straightness.
